# Supplementary material for: Pathogenic Microglia Orchestrate Neurotoxic Properties of Eomes-Expressing Helper T Cells
Source: Cells. 2023 Mar 10;12(6):868. doi: 10.3390/cells12060868 (PMC10047905; doi:10.3390/cells12060868)
Supplement: Supplementary file 1 [file cells-12-00868-s001.zip › cells-2265819-supplementary.pdf]

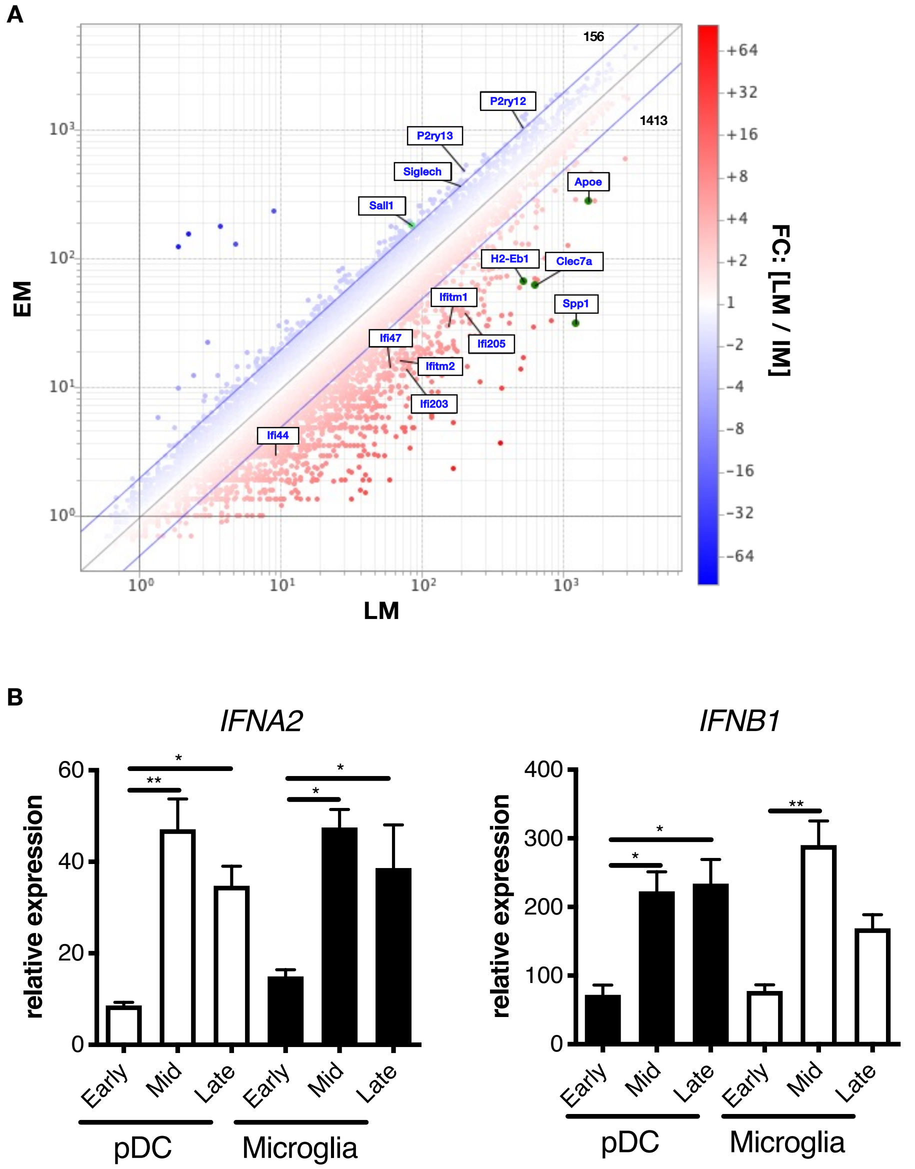

Supplementary Figure S1.
(A) Cx3cr1⁺ CD45 ^{int} microglia were isolated from CNS of EAE Cx3cr1-GFP mice for expression microarray analysis. Gene expression of EM and LM was compared and analyzed using a scatter plot. DEGs were highlighted by fold change ≥2. (B) CNS PDCA-1⁺ B220⁺ pDC and CD45^{int} CD11b⁺ microglia were isolated from early, mid, and late phase of EAE by a FACS Aria II. The expression levels of IFNA2, and IFNB1 were quantified by qPCR. The error bars show mean ± SD. One-way ANOVA test with Dunnett's multiple comparisons test, *, $P<0.05$, **, $P<0.01$.

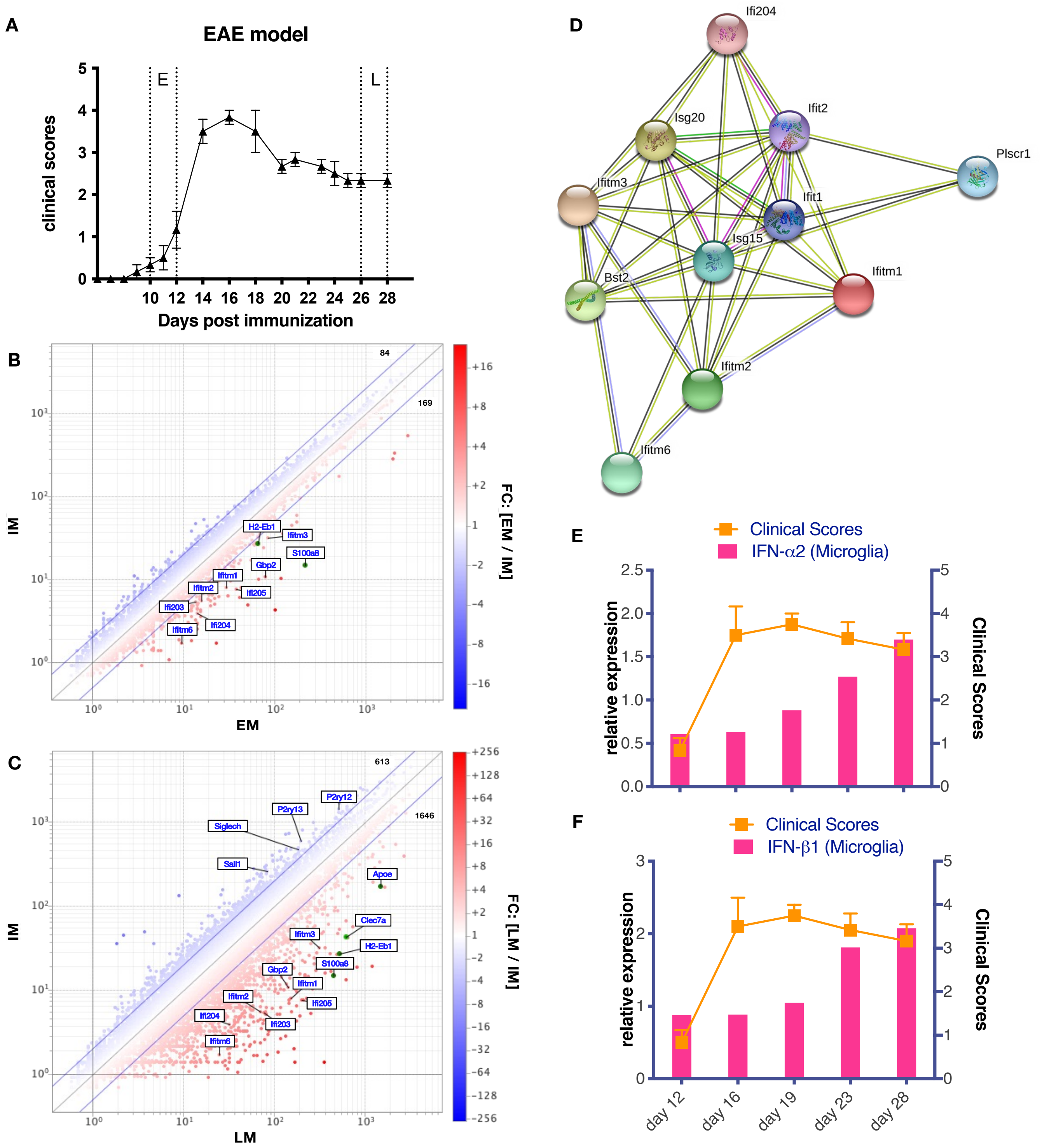
A
EAE model
clinical scores
E
L
Days post immunization
10 12 14 16 18 20 22 24 26 28
B
IM
EM
84
169
H2-Eb1
Ifitm3
S100a8
Gbp2
Ifitm1
Ifitm2
Ifi205
Ifi203
Ifi204
Ifitm6
FC: [EM / IM]
C
IM
LM
613
1646
P2ry12
P2ry13
Siglech
Sall1
Apoe
Clec7a
H2-Eb1
Ifitm3
S100a8
Gbp2
Ifitm1
Ifitm2
Ifi205
Ifi204
Ifi203
Ifitm6
FC: [LM / IM]
D
Ifi204
Isg20
Ifit2
Plscr1
Ifitm3
Ifit1
Isg15
Bst2
Ifitm1
Ifitm2
Ifitm6
E
Clinical Scores
IFN-α2 (Microglia)
relative expression
Clinical Scores
F
Clinical Scores
IFN-β1 (Microglia)
relative expression
Clinical Scores
day 12
day 16
day 19
day 23
day 28

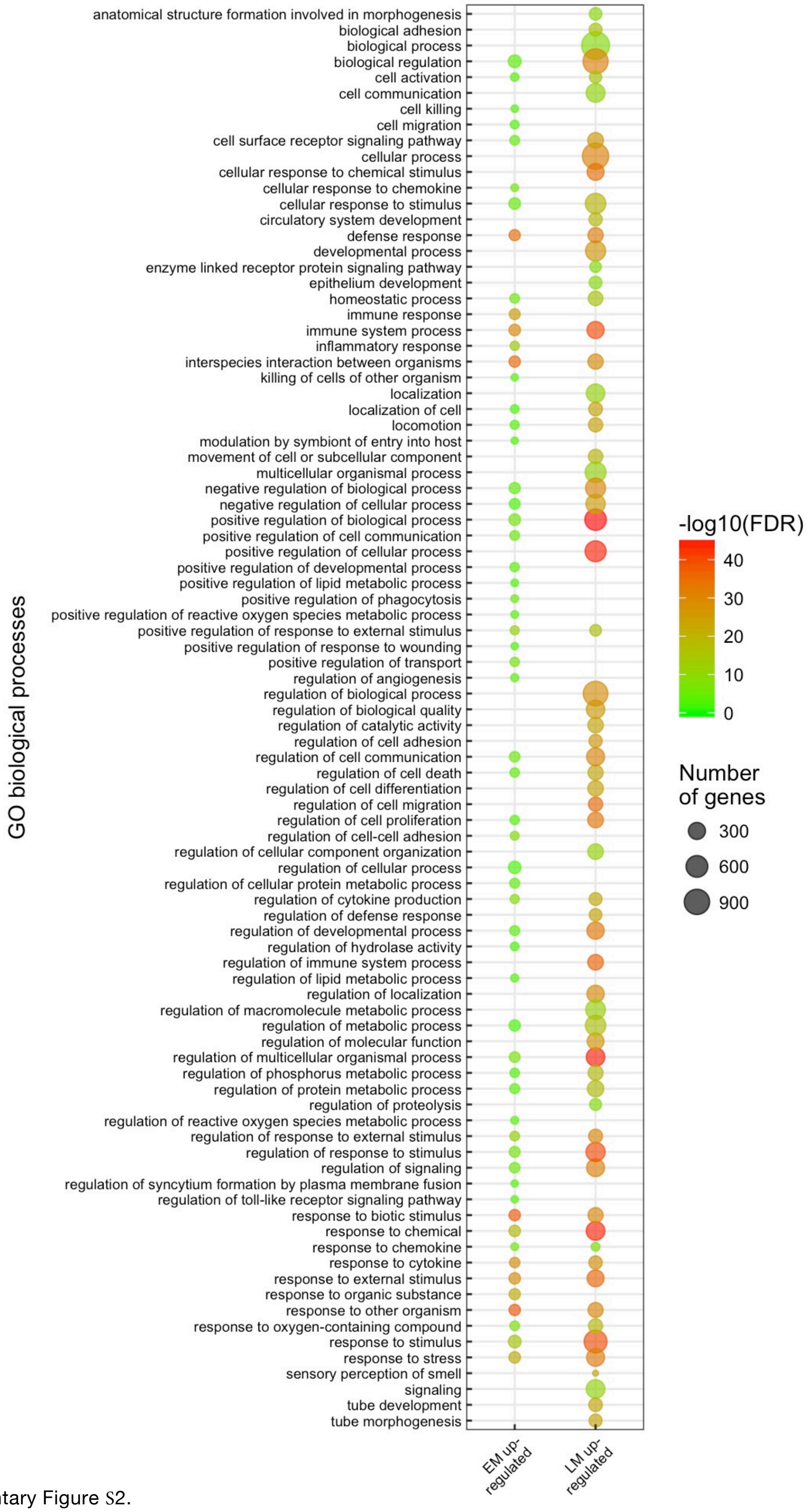

Supplementary Figure S2.
At different time points (intact, early, and late), Cx3cr1⁺ CD45 ^{int} microglia were isolated from CNS of intact or EAE Cx3cr1-GFP mice for expression microarray analysis. Upregulated DEGs (EM vs. IM and LM vs. IM) were run GO analysis using PANTHER. The most significant upregulated 250 GO terms (EM vs IM and LM vs IM) were then screened by using REVIGO to remove redundant GO terms. The representative GO terms from EM vs IM and LM vs IM were selected by the dispensability<0.7. The plot is generated by using ggplot2. The dot size indicates the number of DEGs associated with the process and the dot color indicates the significance of the enrichment (-log10 (FDR-corrected P-values). IM, intact microglia; EM, early EAE microglia; LM, late EAE microglia. See also Fig 2.

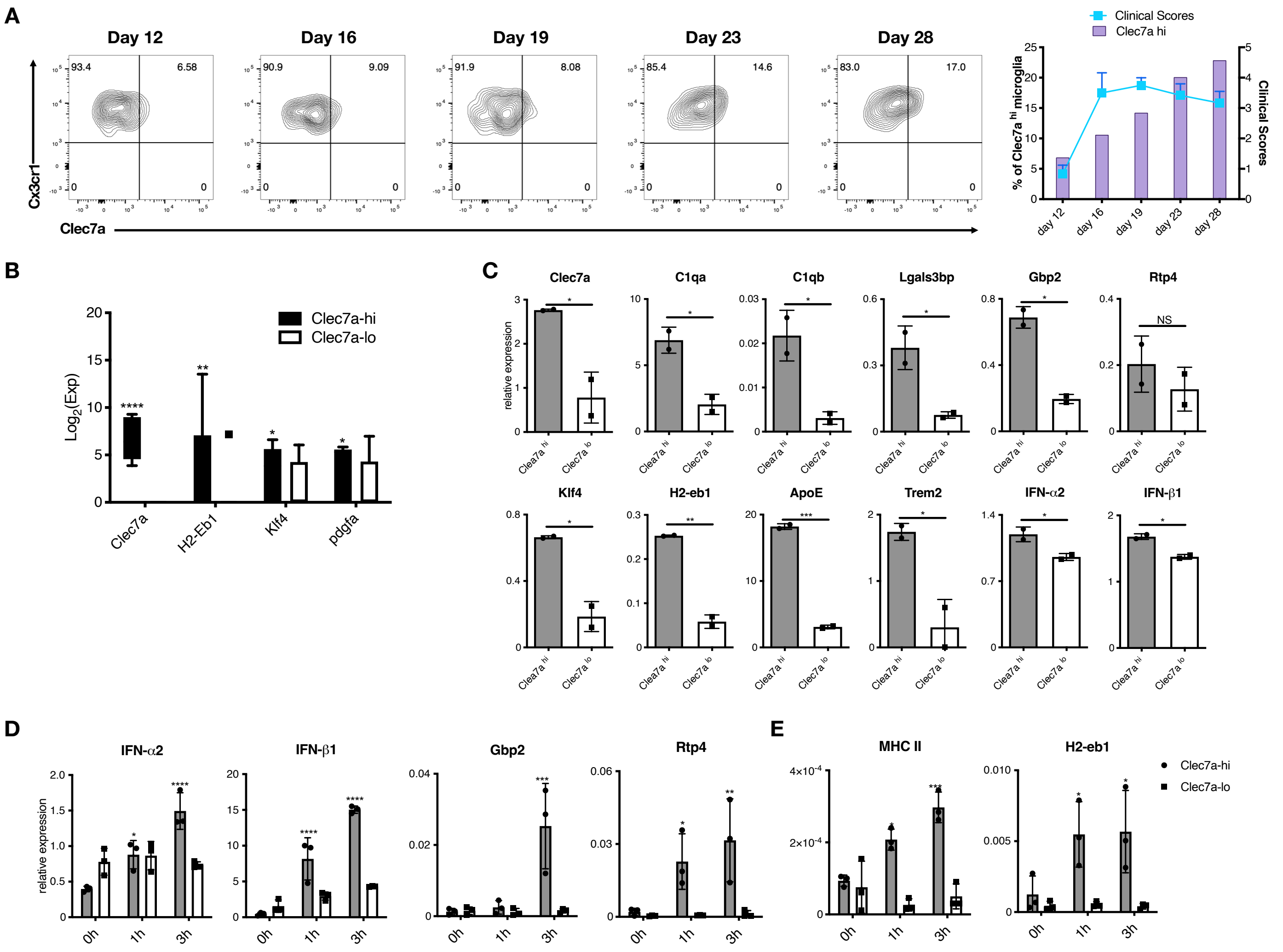

Supplementary Figure S3.

(A) The kinetic of Clec7a in microglia at different time points from EAE Cx3cr1-GFP mice was evaluated by FACS. Represented FACS plots is shown (left) and correlation with clinical score (right). Experiment was repeat performed three times, representative data is shown. (B) Cx3cr1^{+} CD45 ^{int} microglia from early EAE (Day 10-12 post immunization) were divided into Clec7a ^{hi} and Clec7a ^{lo} subsets. The expression of detected genes was shown by a bar graph. Error bars represent the mean ± SD values. Student's t-test; *, *P*<0.05; **, *P*<0.01; ****, *P*<0.0001. (C) Gene expression in sorted Clec7a ^{hi} and Clec7a ^{lo} microglia from late EAE (Day 26-28 post immunization) was determined by qPCR. Error bars represent the mean ± SD values. Student's *t*-test; *, *P*<0.05; **, *P*<0.01; ***, *P*<0.001. (D-E) Sorted Clec7a ^{hi} and Clec7a ^{lo} microglia from intact mice were stimulated with 10 ug/ml Zymosan as indicated time in vitro. Gene expression was determined by qPCR. Error bars represent the mean ± SD values; Statistic difference was determined by comparing with data of 0h, Student's *t*-test; *, *P*<0.05; **, *P*<0.01; ***, *P*<0.001; ****, *P*<0.0001.

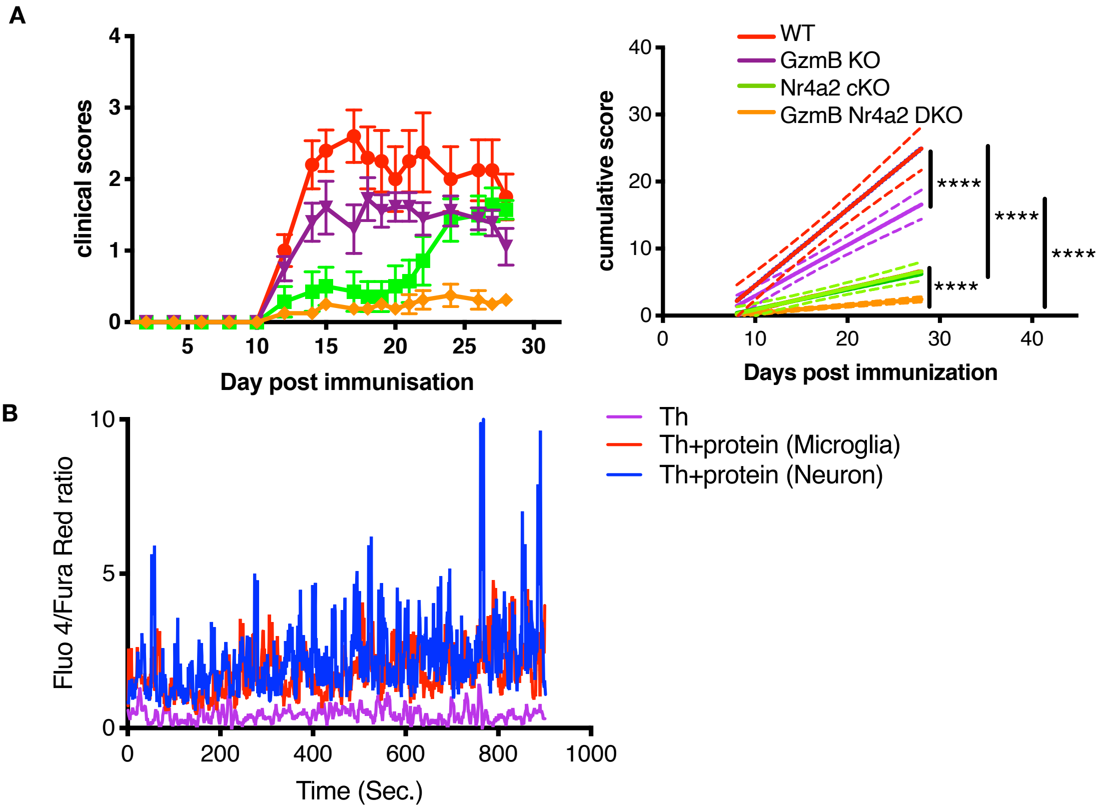

Supplementary Figure S4.
(A) Granzyme B Nr4a2 DKO, Granzyme B KO, Nr4a2 cKO and WT were immunized with MOG_{35-55}. Clinical EAE scores are shown via error bars representing SEM (left). In the right panel, solid lines represent cumulative disease scores; dashed lines indicate the 95% confidence intervals; linear regression analysis; ****, $P<0.0001$. (B) Neuron and microglia were isolated from late EAE mice. Protein then was purified using AllPrep DNA/RNA/Protein Mini Kit (Qiagen). Sorted WT CNS Th cells from peak EAE were stimulated by antigen-loaded (neuron protein / microglia protein) BMDCs. The Ca^{2+} flux of CNS Th cells was measured by a FACS Canto II, the liner graph is shown as ratio of Fluo-4 vs. Fura-Red.

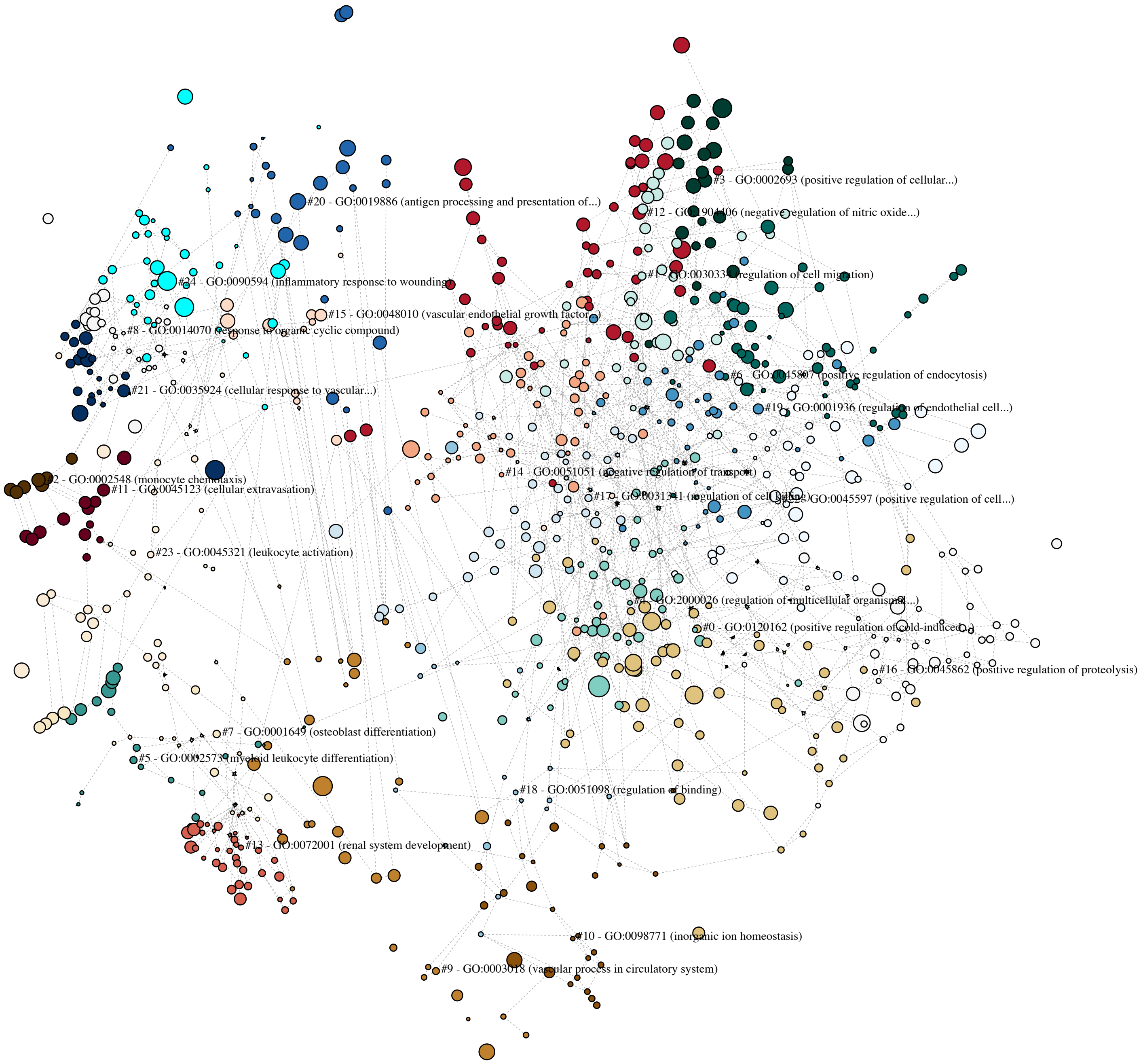

Supplementary Figure S5.

All upregulated DEGs of LM as compared with IM were processed by FunSet. Significant enriched GO terms (FDR<0.01, 871 terms in total) were classified into 25 clusters based on semantic similarity, the terms with the largest average semantic similarity with respect to all terms in the cluster were automatically selected as cluster representatives (medoid terms).

A

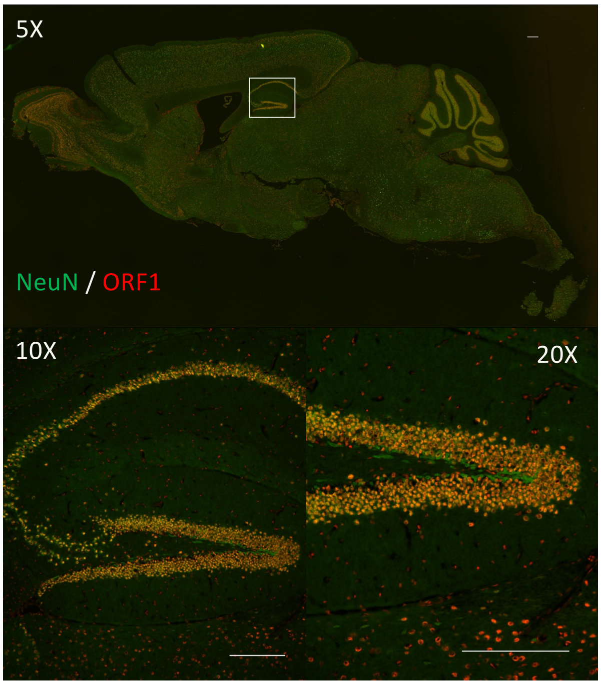

B

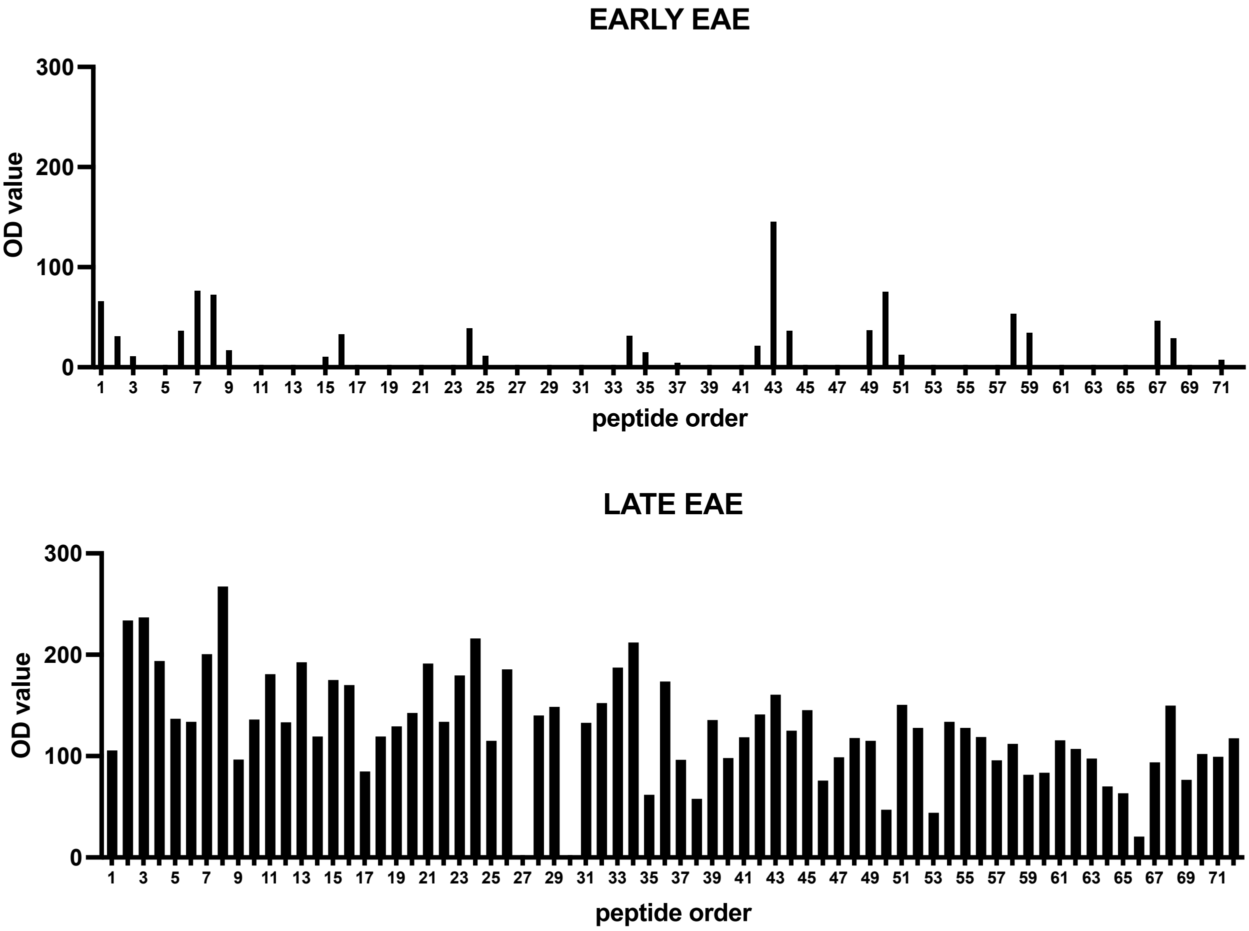

Supplementary Figure S6.
(A) The histology image of late EAE (Day 26-28 post immunization) brain section stained by anti-neuron, anti-ORF1. (B) The sorted CNS Th cells from early (Day 10-12 post immunization) or late EAE (Day 26-28 post immunization) were shortly co-cultured with BMDCs that were pulsed with synthesized ORF-1 peptide library. The Ca^{2+} flux of CNS Th cells was detected and shown as bar graphs determined by OD_{510} value. Two peptides were missed. See also the Method section.

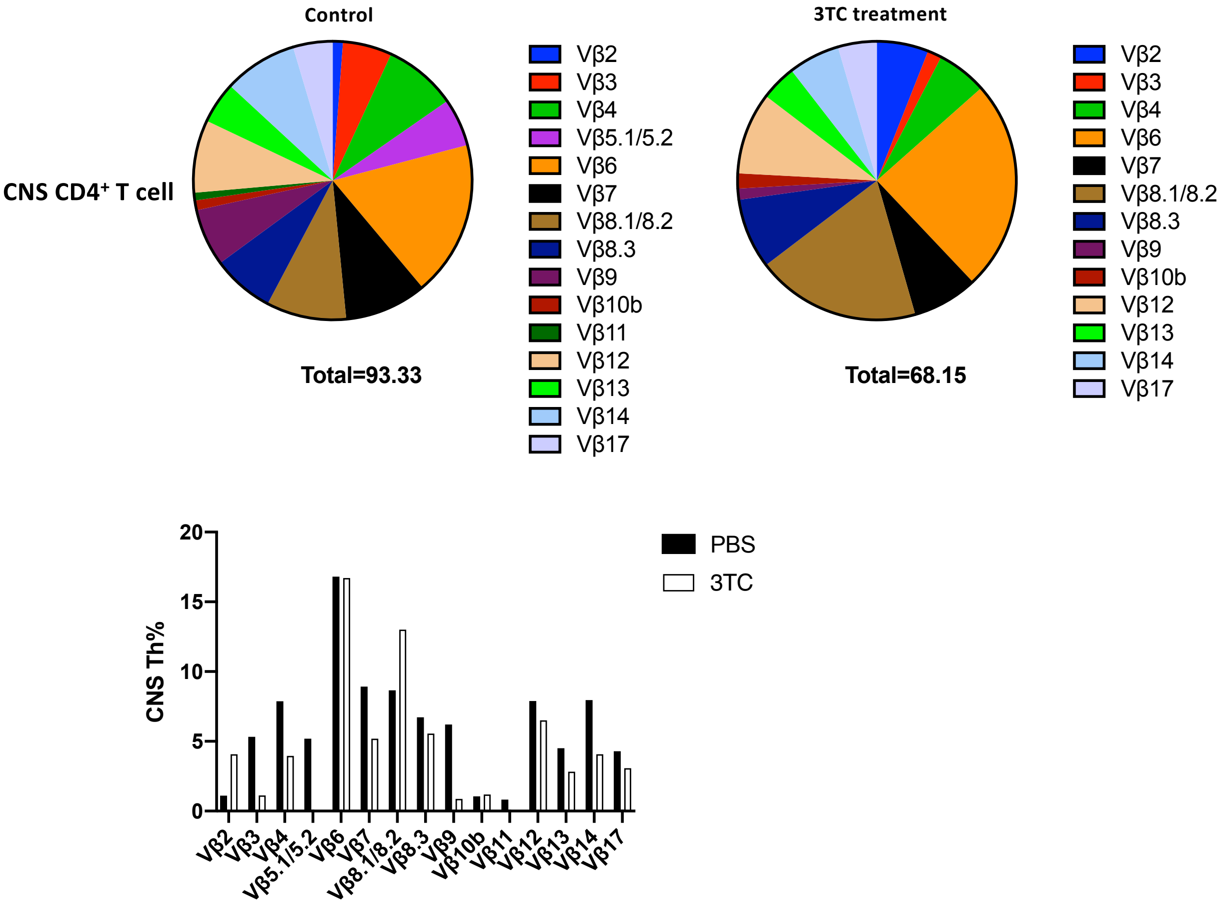

Supplementary Figure S7.

Sorted CNS Th cells from control and 3TC treated EAE mice were surface stained using mouse Vβ TCR screen panel kit. Then intracellular staining was performed against Eomes. Percentages of each Vβ subset among Eomes+ and Eomes- CD4+ T cells are summarized and shown in pie graphs (top) and a bar graph (bottom).

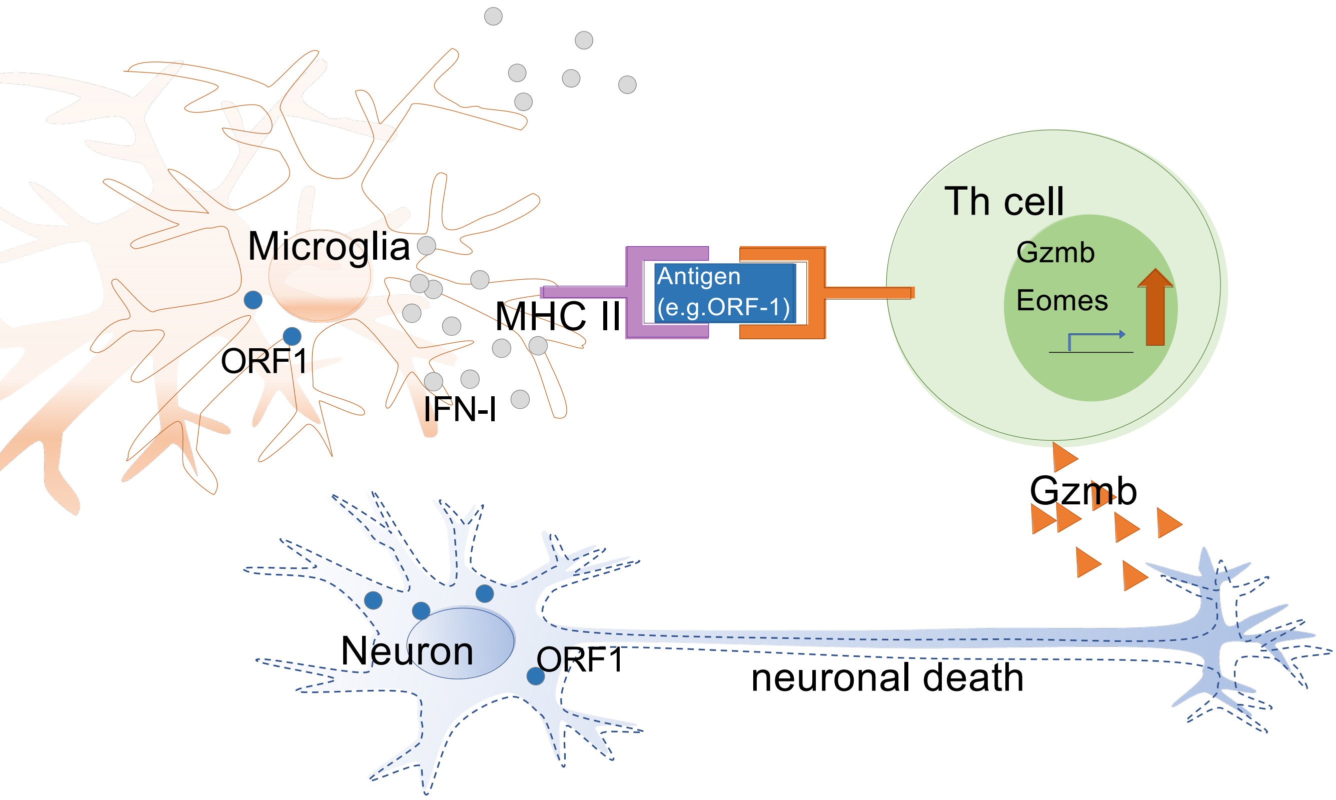

Supplementary Figure S8.
Scheme of suggested hypothetical process of neurodegeneration. See the text also.

LINE-1 ORF1derived peptide

1	MAKGKRRNLTNRNQD	37	NIQEIQDTIRRPNVR
2	RRNLTNRNQDHSPSP	38	QDTIRRPNVRIIGVD
3	NRNQDHSPSPEPSIP	39	RPNVRIIGVDENEDF
4	HSPSPEPSIPTSPSP	40	IIGVDENEDFQLKGP
5	EPSIPTSPSPGNPNT	41	ENEDFQLKGPKIIEE
6	TSPSPGNPNTPENLD	42	QLKGPKIIEEANIFN
7	GNPNTPENLDLDLKA	43	ANIFNKIIEENFPNL
8	PENLDLDLKAYLMMM	44	KIIEENFPNLKNEMH
9	LDLKAYLMMMVEDIK	45	NFPNLKNEMHMNIQE
10	YLMMMVEDIKKDFNK	46	KNEMHMNIQEAYRTP
11	VEDIKKDFNKSLKEI	47	MNIQEAYRTPNRLDQ
12	KDFNKSLKEIQENTA	48	AYRTPNRLDQKRNSS
13	SLKEIQENTAKELQV	49	NRLDQKRNSSRHIII
14	QENTAKELQVLKEKQ	50	KRNSSRHIIIRTSNA
15	KELQVLKEKQENTIK	51	RHIIIRTSNALNKDR
16	LKEKQENTIKQVEVL	52	RTSNALNKDRILKAV
17	ENTIKQVEVLTEKEE	53	LNKDRILKAVREKGQ
18	QVEVLTEKEEKTYKQ	54	ILKAVREKGQVTYKG
19	TEKEEKTYKQVMEMN	55	REKGQVTYKGKPIRI
20	KTYKQVMEMNKTILD	56	VTYKGKPIRITPDFS
21	VMEMNKTILDLKREV	57	KPIRITPDFSPETMK
22	KTILDLKREVDTIKK	58	TPDFSPETMKARRAW
23	LKREVDTIKKTQSEA	59	PETMKARRAWTDVIQ
24	DTIKKTQSEATLEIE	60	ARRAWTDVIQTLREH
25	TQSEATLEIETLGKK	61	TDVIQTLREHKLQPR
26	TLEIETLGKKLSISN	62	TLREHKLQPRLLYPA
27	TLGKKSGTIDLSISN	63	KLQPRLLYPAKLSII
28	SGTIDLSISNRIQEM	64	LLYPAKLSIIIEGET
29	LSISNRIQEMGAEDS	65	KLSIIIEGETKVFHD
30	RIQEMEERISGAEDS	66	IEGETKVFHDKTKFT
31	EERISGAEDSIENIG	67	KVFHDKTKFTHYLST
32	GAEDSIENIGTTIKE	68	KTKFTHYLSTNPALQ
33	IENIGTTIKENGKCK	69	HYLSTNPALQRIITE
34	TTIKENGKCKKILTQ	70	NPALQRIITEKNQYK
35	NGKCKKILTQNIQEI	71	RIITEKNQYKNGNNA
36	KILTQNIQEIQDTIR	72	KNQYKNGNNALEKTRR

Supplemental Table. S1.
ORF-1 peptide library. An ORF-1 peptide library contains 72 overlapping peptides (length: 15 amino acids, offset: 5 amino acids, purity 70%, 1-3 mg/peptide

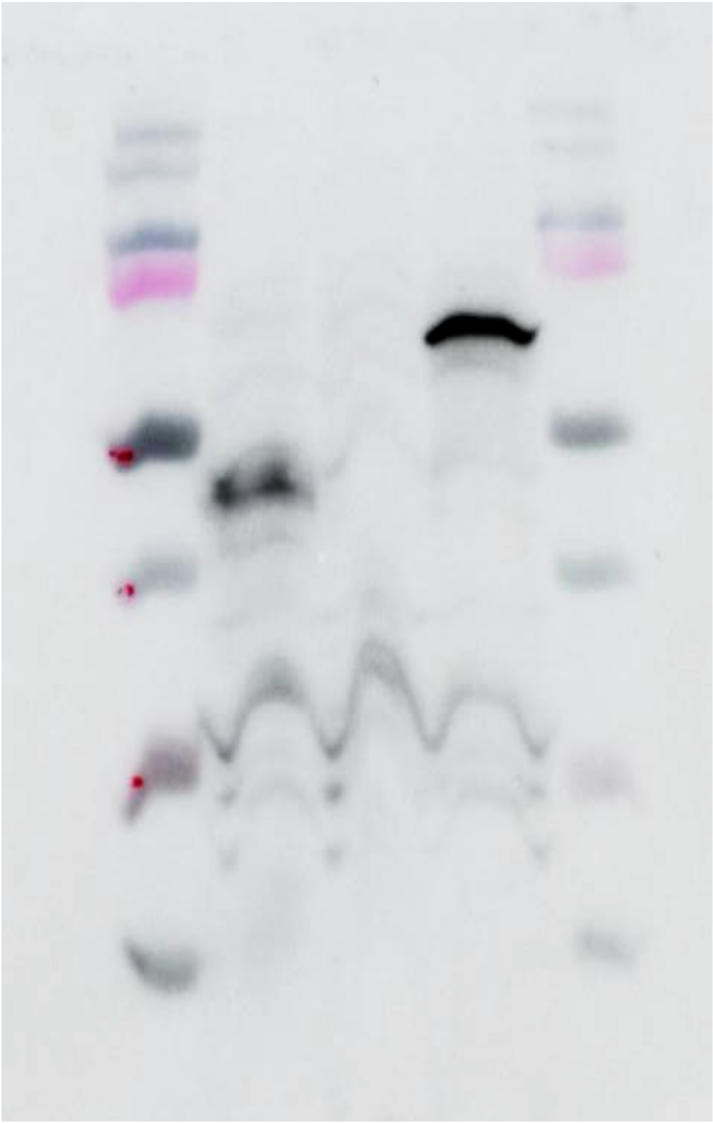

Full unedited gel for Figure 6B
Lane 1: marker
Lane 2: ORF1
Lane 3: Mock
Lane 4: Luciferase
Lane 5: marker
Lane 1 to 3 are cropped and used for fig 6B
